# Lectures on Materia Medica

**Published:** 1884-12

**Authors:** 


### LECTURES ON MATERIA MEDICA.

We have made arrangements with Dr. Cosby, of St. Louis, to furnish the HOMŒOPATHIC PHYSICIAN with notes on Professor Kent's lectures on materia medica. We give, in this number, the notes on *Aceticum Acidum.* During the following year we will give these notes on one or two remedies in each issue. We hope these lectures will be of service to our subscribers. It is not at all probable that these hastily written notes do full justice to Professor Kent's lectures, but as he kindly allows us to use them, we are glad to be able to present them to our readers.

---

